# Catalysis by Hybrid Nanomaterials

**DOI:** 10.3390/molecules26020352

**Published:** 2021-01-12

**Authors:** Leonid M. Kustov

**Affiliations:** 1 N.D. Zelinsky Institute of Organic Chemistry, Russian Academy of Sciences, Leninsky Prospect 47, 119991 Moscow, Russia; lmkustov@mail.ru; 2 National University of Science and Technology MISiS, Leninsky Prospect 4, 119991 Moscow, Russia; 3 Department of Chemistry, M.V. Lomonosov Moscow State University, 1-3 Leninskie Gory, 119991 Moscow, Russia

The state-of-the-art methods of synthesis of nanomaterials in many cases are not able to produce controlled “nano-engineered” nanomaterials that include several sub-nano-sized structurally or functionally organized components. The solution to these problems may be a range of emerging hybrid systems. The currently developed structured and hierarchical hybrid systems are designed for the purposes of reducing energy consumption, increasing the efficiency of processes, and reducing the consumption of expensive components. The use of hybrid nanomaterials provides eco-friendly solutions for environmental problems (purification and remediation of water, air, and soil from harmful compounds including polychlorinated hydrocarbons, chromium, mercury, arsenic, etc.), disposal of waste (lignocellulosic waste biomass, plastic wastes, asphaltenes etc.), and the production of new fuels and products of fine organic synthesis (drugs, polymers), meeting the needs of future generations.

Especially hopes are associated with the use of hybrid materials as catalysts for various processes. Hybrid materials and hybrid nanomaterials differ from composites and nanocomposites:

(1) Each component of the hybrid system itself has a highly organized structure at the molecular and nanoscale level, and the combination of components leads, in turn, to an additionally organized nanostructure constructed from two or more structures of components.
(2) The components of the hybrid system interact with each other to form a chemical structure.
(3) The properties of the hybrid system are determined by the set of properties of the components, while their additivity and synergy may result in new properties that were not observed in the original components.

There are already numerous examples of how combinations of different materials exhibit improved properties compared to individual substances. A hybrid material may consist only of inorganic components or only of organic components. Of particular interest are the materials constructed simultaneously from organic and inorganic components, between which there are regular chemical bonds. As an example, we can consider a material in which, for example, inorganic fibers or nanoparticles are distributed in a polymer or organic matrix and, on the contrary, organic particles and clusters are included in the structure of the inorganic matrix.

There may also exist more complex three- and multicomponent organo-inorganic hybrid systems. The number of organic building blocks is huge, so the number of possible compositions is very large. Accordingly, the number of new hybrid materials is unlimited.

The ultimate result of the interaction between the components of the hybrid system from the point of view of the target function of the material can be (1) averaging of the component properties in a hybrid material, (2) strengthening or improving any function inherent in one of the components by interacting with the second component, (3) adding a property of one material to the list of properties of the second material, (4) synergism of properties, or (5) the appearance of a principally new property in the hybrid material, which was not found in the individual components. The most typical examples of hybrid nanomaterials are organized nanoparticles with a complex composition (metal, oxides, chalcogenides, etc.) and coordination polymers. The nanoparticles can be so-called “core-shell” systems, and the core can be an inorganic particle (metal, oxide, chalcogenide, pnictide, silicon) or an organic particle (polymer globule, organic micelle, dendrimer). The shell can be formed by another metal or any other material. Such “core-shell” nanoparticles are usually encapsulated in a matrix of an oxide, polymer or carbon material, mainly in order to prevent possible coalescence and aggregation of nanoparticles. If the matrix is a porous material, this additionally ensures the transport of other molecules, for example, the substrate of catalytic reactions to hybrid nanoparticles. The matrix itself can be a hybrid nanomaterial, as for example in the case of coordination polymers and metal-organic frameworks. The pore structure of a matrix (zeolite or other crystalline porous material) can also be considered as an additional architecture in the design of such hybrid nanomaterials. There are nanoparticles of one material (metal, oxide, etc.), the surface of which is decorated with sub-nanoparticles of the second material.

Hybrid systems are divided into (1) structurally hybridized materials, (2) chemically hybridized materials with chemical bonds between components, and (3) functionally hybridized materials. The morphology of nanohybrids that can vary from spherical particles, disks, tubes, or filaments to more complex shapes (stars, nested cylinders or cones, etc.) is very important.

The use of hybrid systems in catalysis, adsorption, and environmental protection is particularly important and challenging. The size and shape of nanoparticles (especially of the “core-shell” type or decorated with other sub-nanoparticles) and the interaction with the matrix have a crucial impact on the performance of the catalyst, i.e., its activity, selectivity, and stability. To obtain hybrid systems and materials, the entire arsenal of methods of organic and inorganic chemistry, as well as nanochemistry (the “bottom-up” approach) is widely used:

* template synthesis,
* microwave treatment,
* sol-gel synthesis,
* electrochemical processes (electrophoretic and electrochemical deposition),
* chemical vapor deposition,
* use of ionic liquids and supercritical fluids in the synthesis of hybrid materials.

The use of new hybrid materials includes not only catalysis, but also the following areas:

* gas separation, adsorbents, gas storage,
* sensors,
* energy storage and conversion (solar cells, photodiodes, supercapacitors), energy production from biomass, biogas,
* purification of water, air, and soil from ecotoxicants,
* other applications: materials for space, aviation and special equipment, drug delivery, implants, medical diagnostics, materials for hyperthermia.

The use of ionic liquids is promising for the preparation of hybrid materials, as well as microwave activation. Microwave activation at the preparation stage is effective in mild decomposition of precursors. For a large number of reactions, it was shown that microwave activation in situ also leads to an increase in the efficiency of the nanomaterials. Instead of traditional thermal treatment, resonant microwave activation can be used at the metal reduction stage.

A number of recent publications in Molecules demonstrates the benefits of hybrid nanomaterials, as to their preparation and applications ([1], a review with over 330 references). In particular, the methods providing fine control over the size, morphology, and electronic properties of nanohybrids are of prime importance. Such control can be achieved by using bottom-up synthesis procedures based on colloidal chemistry or atomic layer deposition (ALD) with the focus on mono- and bimetallic materials. Recently, significant efforts were undertaken in enhancing the performance of hybrid nanoparticles by embedding colloidal templates in porous oxide phases or by the deposition of oxide overlayers via ALD. As a recent extension to the latter, the concept of area-selective ALD for advanced atomic-scale catalyst design is put forward.

Organic-inorganic hybrid nanomaterials nowadays represent the most intensively explored class of hybrids. One example of such hybrid systems has been reported recently [2]. Three types of metal oxides (basic MgO, basic-acidic Al_2_O_3_, and acidic-basic Nb_2_O_5_) with mesopores were modified with tris(2-aminoethyl)amine via two methods: (i) direct anchoring of amine onto the metal oxide surface and (ii) anchoring of amine on metal oxide functionalized with (3-chloropropyl)trimethoxysilane. The thus-prepared hybrid materials were shown to exhibit activity in 2-propanol dehydration and dehydrogenation and dehydration and cyclization of 2,5-hexanedione. It was demonstrated that acidic-basic properties of metal oxides as well as the procedure of modification with the amine governed the performance of the materials in Knoevenagel condensation between furfural and malononitrile. MgO-based systems revealed the highest activity in this reaction.

Sometimes, a combination of two different types of matrices gives the best result in terms of the stabilization of metal nanoparticles and their activity/selectivity patterns in catalytic applications. For instance, preparation of porous chitosan–graphene oxide aerogels that combine the high surface area of graphene oxide with the porosity and functionality of chitosan in one matrix provides [3] uniformly sized Pd nanoparticles (~1.7 nm) exhibiting unique catalytic activity in hydrogen generation by ammonium formate decomposition. A turnover frequency above 2200 h^−1^ was reported, which is the highest value ever observed for this process to date. It is extremely important that no formation of CO or CH_4_ was revealed. No Pd leaching occurs during the catalytic tests. Thus, this system is efficient for on-board hydrogen generation from liquid organic hydrogen carriers in transportation.

Environmental applications of hybrid nanomaterials are among the most challenging. Some hybrid ceria-based catalysts were claimed ([4], a review with 114 references.) to demonstrate outstanding catalytic and photocatalytic properties due to the high surface area, enhanced mass transport and diffusion, and accessibility of active sites. Ceria-based mixed oxide systems also provide high oxygen storage capacity, which is crucial for diverse oxidation processes. Of utmost interest are hierarchically organized porous oxide catalysts prepared using template synthetic methods. Catalytic oxidation of volatile organic compounds, soot particulates, and ecotoxic components of exhaust gases, hydrocarbon reforming, water gas shift reaction, and photocatalytic transformations are examples of numerous applications of such ceria-containing nanomaterials. Synthetic or natural compounds can be used as structure-forming templates in the preparation of mixed oxide catalysts based on ceria.

Efficient approaches to fuel production using hybrid nanomaterials based on Ru supported onto halloysite nanotubes via Fischer-Tropsh synthesis was described [5]. Halloysite aluminosilicate nanotubes containing ruthenium nanoparticles were inventively applied as nanoreactors for Fischer–Tropsch synthesis. Modification of the surface of nanotubes with ethylenediaminetetraacetic acid, urea, or acetone azine was carried out in order to improve the ruthenium dispersion. It was found that 3.5 nm Ru particles localized inside the nanotubes manifested enhanced activity and selectivity toward CH_4_, C2–4, and C5+ hydrocarbons produced from CO and H_2_. Modification with ethylenediaminetetraacetic acid provided a methanation catalyst with a 50% selectivity to CH_4_ and C2–4. The application of urea or acetone azine as a modifier led to enhanced selectivity to gasoline-range C5+ hydrocarbons. Localization of Ru inside the nanotubes increased particle stability under reaction conditions.

The majority of hybrid nanomaterials are used in organic synthesis of added value products. Metal organic frameworks, covalent organic frameworks, hydrogen-bonded organic frameworks, and other types of coordination polymers represent the whole universe of novel organic-inorganic or purely organic hybrids and include over 100,000 known and characterized structures, with their compositions containing over 80 elements of the periodic table of elements. They are very perspective in organic syntheses (see, for instance, [6]). Metal-organic frameworks (MOF) containing lanthanum(III), cerium(III), neodymium(III), europium(III), gadolinium(III), dysprosium(III), and holmium(III) and 1,3,5-tris(4-carboxyphenyl)-2,4,6-trimethylbenzene as the precursor of the ligand were synthesized using the solvothermal method. Permanent porosity and thermal stability up to 500 °C are the distinguished properties of these MOF materials. These Ln-MOFs exhibit excellent catalytic activity in acylation of 2-naphthol.

Inorganic hybrid nanoparticles based on iron were tested in partial oxidation, in particular, oxidation of 1-phenylethanol to acetophenone [7]. The nanoparticles with sizes ranging from 0.1 to 1 micron were prepared via wet and ball-milling methods. In the case of the former method, semi-spherical magnetic nanoparticles were produced, whereas the ball-milling method gave nonmagnetic micron-scale needles and rectangles. Accordingly, the catalytic activity of two types of materials in the oxidation of 1-phenylethanol to acetophenone under conventional heating, microwave irradiation, ultrasound treatment, and under an oscillating magnetic field of high frequency (induction heating) was very much dependent on the method of synthesis. Thus, the magnetic semi-spherical nanoparticles demonstrate lower activity, while the needle/rectangle samples provide a better acetophenone yield (83%). A very important result of the study is a 4-fold increase in the yield under induction heating compared to conventional heating. The further advantage of the system is the possibility of the magnetic separation of the catalyst, which luckily turned out to be more active than the non-magnetic catalyst.

Diverse hybrid catalysts can find applications in renewables valorization [8]. Biomass is the source of carbon to replace petroleum, natural gas, and coal in production of renewable fuels as well as added-value organic products. 

Inorganic and mixed organic-inorganic hybrid nanomaterials are perspective for photocatalytic reactions [9]. Porous V_2_O_5_/TiO_2_ nanoheterostructure films with atomic Ti/V ratios ranging from 4:1 to 1:2 were prepared using a sparking method and demonstrated enhanced visible-light absorbance resulting in high photocatalytic activity in degradation of methylene blue in water. It is the existence of the nanoheterostructure and the significant concentration of V^4+^ ions that govern the activity pattern of the nanocatalyst.

Another example is the Cu_2_O nanostructure decorated with bismuth oxide [10] that showed enhanced phototocatalytic activity in hydrogen production from water under visible light.

Magnetically recoverable TiO_2_/SiO_2_/γ-Fe_2_O_3_/rGO nanohybrid revealed a significantly enhanced UV-visible light photocatalytic activity in degradation of methylene blue [11]. The degradation efficiency increased to 84% under UV light, which was accounted for by the higher adsorption of dye on the surface of the nanomaterial.

The photocatalytic activity of iso-propylamine-based lead chloride perovskite, which is now the priority material for solar cells, has been revealed towards depolymerization of lignin under UV light at room temperature and at 90 °C [12]. The depolymerization extent reached 90–98% for 60 min. The activation energy for depolymerization was 15 kJ/mol, which is much lower than the reported values of about 60 kJ/mol. The major products were phenols and benzene.

Diverse hybrid nanomaterials are widely used in membranes [13], including mixed matrix membranes. Ceramic membranes with enhanced photocatalytic activity prepared using the sol-gel method under solvent-free conditions exhibited efficient degradation of methylene blue. 

Finally, hybrids are among the leaders in biocatalysis applications because they allow controllable immobilization of enzymes. The high activity of biosilicified lipases immobilized onto a sol-gel hybrid material in esterification of valeric acid to produce esters has been demonstrated [14]. Conventional heating and microwave irradiation were compared. Ethyl valerate yields reached 85% (15 min). 

To conclude, the future of nanohybrids looks bright and new, yet unexplored materials of this kind will remain the priority in the next decades.
